# Low defibrillation threshold in pediatric patients with subcutaneous implantable cardioverter defibrillator: Implications for a smaller generator?

**DOI:** 10.1016/j.hrcr.2026.03.021

**Published:** 2026-04-25

**Authors:** Jolien A. de Veld, Willeke van der Stuijt, Shari Pepplinkhuizen, Nico A. Blom, Lonneke Smeding, Kirsten M. Kooiman, Reinoud E. Knops

**Affiliations:** 1 Amsterdam UMC location University of Amsterdam, Heart Center, Department of Cardiology, Amsterdam Cardiovascular Sciences Heart failure & Arrhythmias, Amsterdam, The Netherlands; 2 Department of Pediatric Cardiology, Amsterdam University Medical Center, University of Amsterdam, The Netherlands; 3 European Reference Network for Rare and Low Prevalence Complex Diseases of the Heart (ERN GUARD-Heart), Amsterdam, The Netherlands

**Keywords:** Subcutaneous ICD, Transvenous ICD, Pediatrics, Defibrillation threshold, Ventricular arrhythmia

**Key Teaching Points**

• Subcutaneous implantable cardioverter-defibrillator (S-ICD) implantation in pediatric patients is feasible, but the generator size poses a challenge in small children because of limited anatomical space.
• In pediatric patients, the S-ICD is able to convert 100% of induced ventricular arrhythmias with a single 30 J shock, marking the first step toward a smaller generator size.
• Strategies should be implemented to mitigate the risk of defibrillation failure following substantial growth or weight gain in pediatric patients during follow-up.

## Introduction

The subcutaneous implantable cardioverter-defibrillator (S-ICD) is a completely extra-thoracic device developed to overcome complications associated with transvenous leads.^1^ Owing to this design, the S-ICD requires a higher energy output to ascertain effective defibrillation, compared with the transvenous ICD (TV-ICD). The default shock output of the S-ICD was set at 80 J, which was based on 2 studies conducted in the early years of S-ICD therapy.^1^ This resulted in a generator which is twice as large as the TV-ICD. However, later research has shown a mean defibrillation threshold (DFT) of 29 ± 12 J at S-ICD implantation.^2^ In addition, at generator replacement, a median DFT of 20 J for patients with a body mass index (BMI) ≤25 and 30 J for patients with a BMI >25 was reported.^3^

The large generator of the S-ICD results in more post-operative pain.^4^^,^^5^ Although this does not result in a significant difference in overall quality of life (QoL) compared with patients with TV-ICD, patients with S-ICD expect improvement of their QoL by ICD deactivation or extraction, which is mainly driven by discomfort and pain from the S-ICD generator pocket.^6^^,^^7^ These findings underline that a size reduction is desired.

Owing to the smaller body size of pediatric patients, it is likely that the DFT in children will be lower. In addition, the S-ICD appears more prominently on their bodies. This makes them more susceptible to lead- and pocket-related complications.^8^^,^^9^ Besides this, these patients often have little subcutaneous fat tissue and a small latissimus dorsi muscle, which makes it hard to properly cover the generator. Furthermore, their sternum or thorax may be too small for the lead and generator. These characteristics sometimes require a modified implant technique in small children. This emphasizes the need and possibilities for a smaller generator in this population.

In this case series, we present data from pediatric patients who underwent S-ICD implantation and subsequent step-down defibrillation testing to assess the DFT, defined as the lowest tested energy at which the S-ICD terminated an induced ventricular arrhythmia with 1 single shock. In addition, we provide tools to help physicians overcome challenges during S-ICD implantation in pediatric patients.

## Case Series

This case series describes 7 pediatric patients undergoing S-ICD implantation. The age of these patients ranged from 8 to 17 years old, with a height of 1.29–1.78 meters and a weight of 27.4–65.4 kg. Four out of 7 patients received their ICD for a hypertrophic cardiomyopathy and 3 due to an inherited arrhythmia syndrome. Two of these patients were female and 4 received their ICD for primary prevention. The characteristics per case are presented in Table 1.

**Table 1:** Characteristics per patient

Patient number	Sex	Age	Height and weight	ICD indication	Primary or secondary prevention	Defibrillation threshold	First shock impedance	PRAETORIAN score	Follow-up duration
1	M	12	1.57 m 46 kg BMI 18.7 kg/m^2^	HCM	Primary	10 J	70 ohm	NA due to right parasternal implantation.	5 years
2	F	15	1.75 m 52.7 kg BMI 17.2 kg/m^2^	Inherited arrhythmia syndrome	Secondary	20 J, no 10 J shock performed	55 ohm	NA due to unavailability of lateral radiograph	4 years, 8 months
3	F	8	1.38 m 33.5 kg BMI 17.6 kg/m^2^	HCM	Primary	10 J	79 ohm	30 points	4 years
4	M	17	1.78 m 61 kg BMI 19.3 kg/m^2^	Inherited arrhythmia syndrome	Primary	20 J	50 ohm	30 points	3 years
5	M	15	1.74 m 65.4 kg BMI 21.6 kg/m^2^	HCM	Primary	10 J	57 ohm	30 points	2 years, 7 months
6	M	11	1.56 m 49 kg BMI 20.1 kg/m^2^	HCM	Secondary	20 J	87 ohm	30 points	2 years, 1 month
7	M	8	1.29 m 27.4 kg BMI 16.5 kg/m^2^	Inherited arrhythmia syndrome	Secondary	30 J	79 ohm	NA due to right parasternal implantation.	3 months
**Overall results**	**29% F**	**12 ± 3**	**1.58 ± 0.18 m47.9 ± 12.7 kg** **BMI 18.7 kg/m^2^**	**57% HCM**	**43% secondary prevention**	**17 J (95% CI 10–24)**	**68 ± 13 ohm**	**PRAETORIAN score 30 for all patients with eligible X-rays**	**35 months (IQR 25–55)**

BMI = body mass index; CI = confidence interval; F = female; HCM = hypertrophic cardiomyopathy; M = male; ICD = implantable cardioverter-defibrillator; NA = not available; IQR = interquartile range.

### Pre-operative screening and consultation

**Figure 1:**
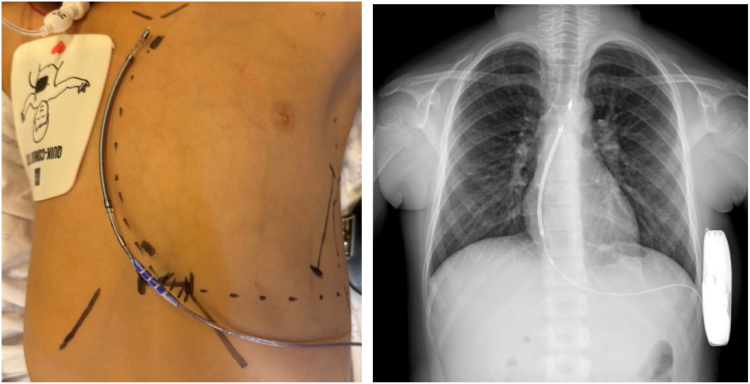
Curved lead position in a very small patient. Before incision, the implantation location is marked to achieve optimal positioning. In this patient the lead is implanted in a curved position, to accommodate for future patient growth.

All patients underwent pre-operative S-ICD screening and consultation. During the consultation meeting, the S-ICD was compared with the TV-ICD and risks and benefits were discussed according to literature available at that time, including the larger size of the S-ICD generator. Besides this, patients were screened for S-ICD eligibility. In case of a very small patient, the lead was placed in a curved position to accommodate for expected growth. During screening, the optimal position of the lead was determined, and the implant position was marked (Figure 1). Finally, the skin was examined to ensure that there was sufficient subcutaneous tissue for appropriate generator placement.

### Implantation

**Figure 2:**
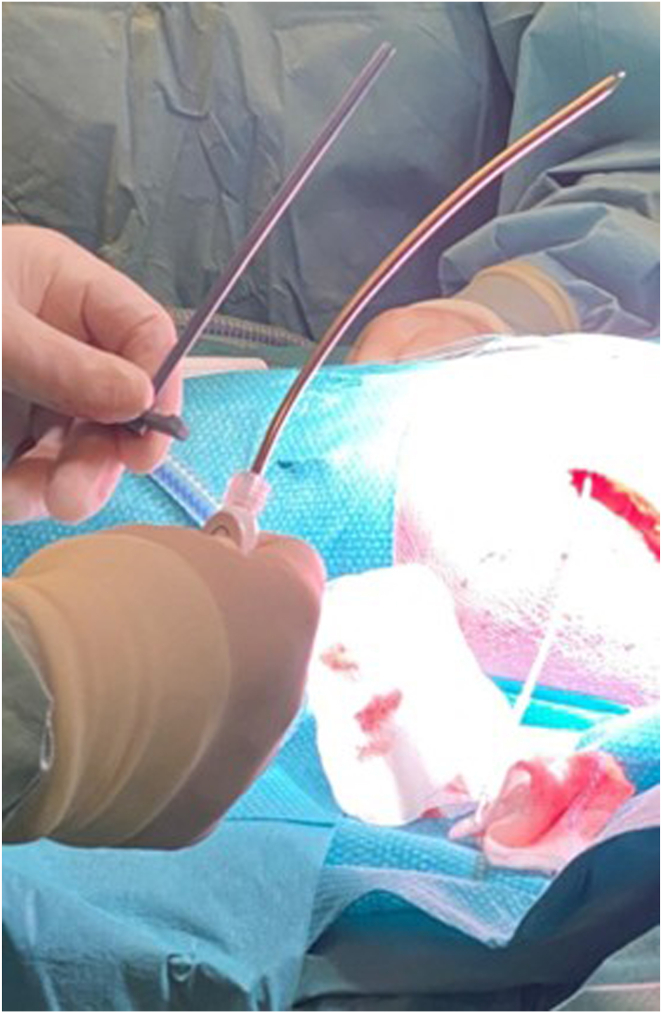
The manually pre-shaped curved tunneling tool. For curved lead implantation, the tunneling tool is manually bent.

Implantation commenced with placement of the lead. In patients with a sternal length >16 cm (measured from xyphoid to jugulum), the lead was placed in the standard straight position and tunneled on the sternum through the substernal incision. In 2 very small patients with a short sternal length, the lead was implanted in a slightly curved position using a manually pre-shaped tunneling tool (Figure 2). In the curved position the coil is extended right parasternal which may, if any, have a lowering effect on DFT and keep the sensors in the standard pre- or parasternal position. Whenever possible, the generator was placed in the intermuscular position. Special care was taken to ensure that the device did not protrude posteriorly, to prevent patient discomfort when leaning back. Besides this, a slightly large pocket was created to prevent decubitus. Lastly, traction on the skin during pocket closure was avoided.

### Defibrillation testing

**Figure 3:**
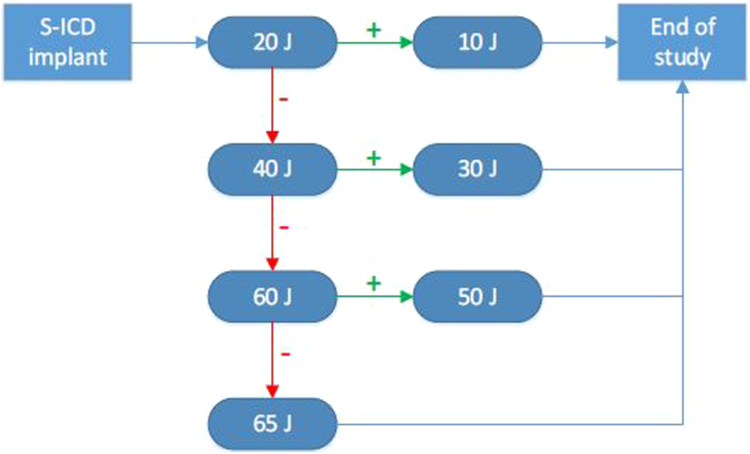
Step down protocol to determine the defibrillation threshold. Binary search defibrillation protocol. + Indicates the induced ventricular arrhythmia was successfully terminated. – Indicates a failed conversion. If the patient failed to convert at 65 J, the patient was treated according to standard-of-care for a patient with defibrillation-test failure. This usually includes repositioning of the device or performing a chest radiograph to determine if there is air trapped around the coil or generator. S-ICD = subcutaneous implantable cardioverter-defibrillator.

**Figure 4:**
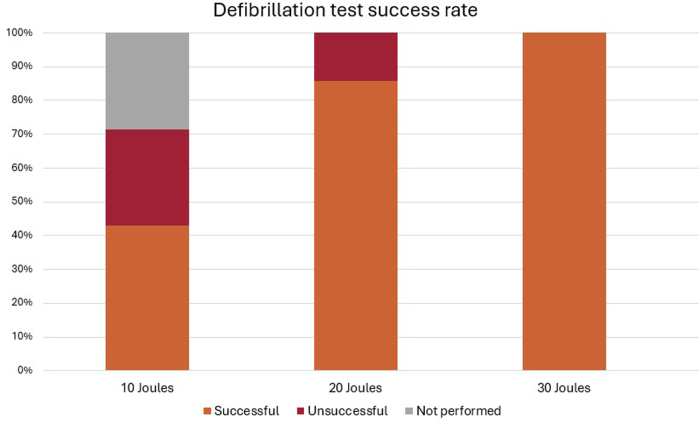
Defibrillation test success at different energy levels. On the y-axis the percentage of successful conversions is displayed, with the tested energy on the x-axis.

All patients were tested according to a pre-specified step-down defibrillation testing protocol (Figure 3), which is comparable to the defibrillation testing protocol used in the first S-ICD studies by Bardy et al.^1^ To minimize the number of shocks per patient, step-down testing was performed with 10 J intervals. This makes the goal of this protocol to verify shock efficacy at various energy levels, rather than finding the precise minimum DFT. Before implantation, patients and/or their parents provided written informed consent prior to participation in this procedure, and the protocol was approved by the local Institutional review board. The ventricular arrhythmia was induced using a 50 Hz pacing burst, whereafter the S-ICD delivered a shock in standard polarity. If the S-ICD failed to convert the induced arrhythmia, an external shock was delivered. As displayed in Figure 4, 6 patients had a successful conversion with a single 20 J shock. In 5 of these patients, ventricular fibrillation (VF) was induced once more and a 10 J shock was administered by the S-ICD. This shock was successful on 3 of 5 patients. In 1 patient, the 10 J defibrillation test was not performed due to multiple failed inductions preceding the 20 J shock, whereafter the implanting physician decided to abandon the testing protocol. There was 1 patient in whom the 20 J shock was unsuccessful. Interestingly, this was the smallest patient in our cohort, with a height of 1.29 m and a weight of 27.4 kg. In this patient, 2 additional defibrillation tests were performed with 30 J and 40 J, which were both successful. The mean DFT in these 7 patients was 17 J (95% confidence interval 10–24). All ICDs were programmed at the default shock output of 80 J after implantation.

### Follow-up

**Figure 5:**
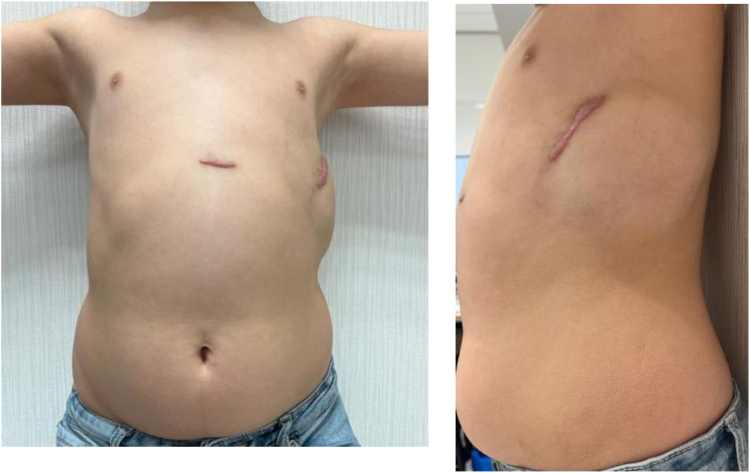
S-ICD in a small patient 3 months after implantation. Lateral and anterior view of a small patient 3 months after S-ICD implantation. The figure shows the large size of the generator in relation to the thorax. S-ICD = subcutaneous implantable cardioverter-defibrillator.

After a median follow-up of 35 months (IQR 25–55), no S-ICD related complications were reported. Two months after implantation, every patient had a follow-up visit to evaluate the implantation result after wound healing (Figure 5). Six patients did not receive appropriate therapy from their ICD, whereas 1 patient with hypertrophic cardiomyopathy had 5 arrhythmic episodes during a follow-up of 25 months. All arrhythmias were VF during exercise and were terminated by the S-ICD. In 4 episodes, the first shock was successful, whereas the second shock terminated the arrhythmia in the remaining episode. The high voltage impedance ranged from 70–83 ohms. No inappropriate shocks were reported.

## Discussion

To our knowledge, this is the first case series reporting on step-down defibrillation testing to explore the DFT in pediatric patients after S-ICD implantation. The main finding is a 100% conversion success with 30 J, indicating that with a 10 J safety margin, an S-ICD that delivers a 40 J shock would be sufficient. These results mark the first step in creating a smaller S-ICD generator designed for pediatric patients, but an expansion of the sample size is required to draw any definitive conclusions.

An earlier study showed low DFT in pediatric patients implanted with a transvenous or abdominal ICD.^10^ In the cohort of 20 patients with weight ≤60 kg, the median DFT was 7 J and 19 of 20 patients had a successful conversion with ≤15 J. This DFT is lower than reported in adults, where the mean DFT reportedly ranged from 11.3–17 J depending on the implantation location of the can.^11^ In contrast, another study reported an inadequate defibrillation safety margin in 13.6% of pediatric patients, which is higher than found in adult studies.^12^ The authors do not present a clear reason for this discrepancy, other than that factors associated with an inadequate defibrillation safety margin, such as the absence of ischemic heart disease, were more prevalent in their study cohort.

For S-ICDs, step-down defibrillation testing has only been described in adults, with a mean shock energy of 29–33 J at implantation^2^^,^^13^ and 27.4 J at generator replacement.^3^ DFT is lower in patients with a BMI ≤25, whereas fat underlying the S-ICD coil or generator causes a higher DFT.^14^ In our cohort, the mean DFT was 17 J, which is markedly lower than the DFT in adults. Pediatric patients usually have a lower BMI and less adipose tissue compared with adults, which explains their lower DFT. In our cohort, the mean BMI was 18.7 kg/m^2^, which is markedly lower than the mean BMI in previous research, which was 22.5 kg/m^2^. This may explain why our data shows a 100% success rate at 30 J compared with 82% in adults with BMI <25. Furthermore, the close proximity of the lead and generator in relation to their left ventricle because of their small body size might explain their lower DFT.

### Implantation strategy in small patients

There are multiple ways to improve S-ICD implantation in small patients, to minimize the risk of complications and discomfort. In general, it is important to evaluate every patient individually to estimate whether the patients’ body size is sufficient for an S-ICD. A previous case report of S-ICD implantation in a 7-year-old patient with a height of 120 cm and weight of 17 kg, showed that S-ICD implantation can be performed in very small patients.^15^ However, this case report does not provide any visual material on the S-ICD generator position after implantation other than a posteroanterior chest radiograph. In contrast, a case series of 11 patients aged 12–17 years published multiple photographs of the S-ICD generator after implantation.^16^ Even though no complications were reported in these patients, the photographs demonstrate the large generator size in slim pediatric patients.

### Future perspectives

We found a 100% shock efficacy with 30 J in pediatric S-ICD patients, but it is unknown if this low threshold will extend beyond these 7 patients and during further follow-up. Pediatric patient growth will result in a larger distance between the generator and coil, possibly causing a higher DFT. Besides this, significant weight gain has been linked to defibrillation failure.^17^ The high rate of defibrillation test success at lower shock energies in adult patients suggests that defibrillation with 30 J is likely to remain effective in our cohort once they reach adulthood. To confirm this, repeat testing with 30 J should be considered after patients have completed growth. If a smaller S-ICD generator were to be developed, strategies should also be implemented to mitigate the risk of defibrillation failure following substantial growth or weight gain in pediatric patients.

An alternative to downsizing the S-ICD generator is implantation of an EV-ICD (medtronic) or extravascular-ICD (Adtacor + abbott).^18^^,^^19^ Successful implantation of an EV-ICD in a 2-year-old has already been documented.^20^ While the intercostal delivered extravascular-ICD lead is not yet commercially available, it may offer a future alternative. Both the EV-ICD and extravascular ICD lead systems deliver 40 J shocks and have demonstrated efficacy in terminating ventricular arrhythmias in adults, potentially offering an advantage over the S-ICD because of their smaller size.

## Limitations

This case series is limited by the low number of patients. ICD implantation in pediatric patients is scarce, and parents are not always willing to let their child participate in scientific studies. Nevertheless, this is the only cohort to date describing step-down defibrillation testing in pediatric patients with an S-ICD, which makes our findings unique. Lastly, because of the probabilistic nature of defibrillation testing, the first failed test may not always represent the true DFT. To determine the DFT more reliable, multiple inductions per energy level should be performed. However, to limit the number of inductions for our patients, we did not test multiple times in case of failure in our cohort.

## Conclusion

Our data shows that in pediatric patients the S-ICD is capable of terminating 100% of ventricular arrhythmias with a 30 J shock with a mean DFT of 17 J, which could be the first step toward a smaller generator. Our findings are intended to form a basis for further research in this field. In addition, studies with additional defibrillation tests during follow-up are needed to ascertain effectiveness of lower energy shocks after these patients reach adulthood.

## Disclosures

K.M.K. reports consultancy fees from Boston Scientific. R.E.K reports consultancy fees and research grants from Abbott, Boston Scientific, Medtronic, and Cardiac and has stock options from AtaCor Medical Inc. The other authors report no conflicts of interest.
